# Watchful waiting versus totally extraperitoneal (TEP) hernia repair for occult inguinal hernia and pain (EFFECT trial)—a multicenter, non-inferiority, randomized controlled trial

**DOI:** 10.1007/s00464-025-11681-w

**Published:** 2025-05-22

**Authors:** R. R. Meuzelaar, E. J. M. M. Verleisdonk, A. H. W. Schiphorst, F. P. J. den Hartog, P. J. Tanis, J. P. J. Burgmans, EFFECT study group

**Affiliations:** 1 Department of Surgery, Erasmus Medical Center, Rotterdam, The Netherlands; 2 Department of Surgery, Diakonessenhuis, Utrecht, The Netherlands

**Keywords:** Groin pain, Occult inguinal hernia, Watchful waiting, TEP repair, Quality of life, Crossover

## Abstract

**Background:**

Current international guidelines offer no specific recommendations for managing occult inguinal hernias with groin pain, often resulting in unnecessary repairs. This randomized controlled trial (RCT) evaluated whether watchful waiting (WW) is non-inferior to totally extraperitoneal (TEP) repair in this distinct patient population.

**Methods:**

From December 29, 2017, to March 4, 2022, this multicenter, non-inferiority RCT screened all adult patients with unilateral groin pain (numeric rating scale [NRS] ≥ 1) without a clinically evident inguinal hernia. Patients allocated to the WW arm were treated with rest, analgesics, or physiotherapy, while those assigned to surgery underwent TEP repair. The sample size was 80 patients per arm (non-inferiority margin: 0.75 NRS; 1-sided alpha: 0.025; beta: 0.10; loss to follow-up: 10%). The primary outcome was the mean NRS difference between baseline and 3 months of follow-up, measured at rest and during exercise, and analyzed using a mixed-effects model. Total follow-up was 12 months. Secondary outcomes included quality of life, patient satisfaction, and crossover rate.

**Results:**

From a total of 99 patients, 85 patients were included in the study (WW: 49; TEP: 36). The analysis showed a mean difference of 0.644 (97.5% CI: − 0.321 to 1.610) for pain at rest and 0.806 (97.5% CI: − 0.402 to 2.014) for pain during exercise. Crossover from WW to TEP occurred in five patients (10%). Secondary outcomes were similar between the groups up to 3 months.

**Conclusion:**

This trial failed to demonstrate non-inferiority of WW compared to TEP repair for pain relief at 3 months post-intervention in patients with groin pain and an occult inguinal hernia. However, this result does not confirm that WW is inferior, as secondary outcomes were comparable up to 3 months and upfront TEP repair carried a risk of overtreatment. Therefore, a WW strategy for at least 3 months may be justified as a diagnostic tool to determine which patients may benefit from surgery.

**Graphical Abstract:**

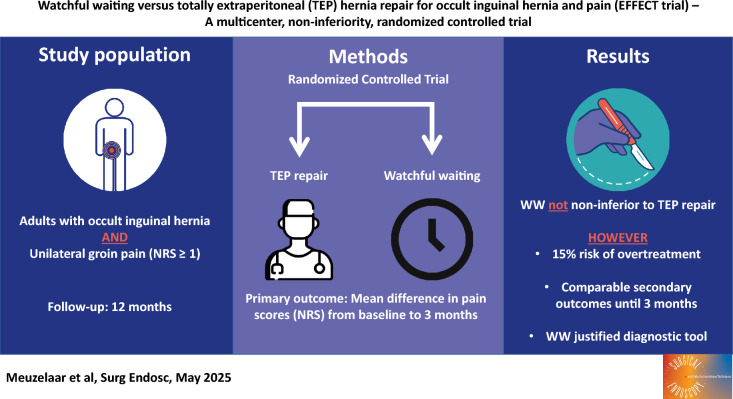

**Supplementary Information:**

The online version contains supplementary material available at 10.1007/s00464-025-11681-w.

Groin pain in adults is a frequent complaint encountered in surgical practice and is often associated with inguinal hernias [1]. However, the diagnosis and treatment of patients presenting with groin pain in the absence of clinical signs of an inguinal hernia can be challenging [2, 3]. Current international guidelines advise further investigation in these patients using ultrasonography and, if inconclusive, magnetic resonance imaging (MRI) [4, 5]. When additional imaging reveals an inguinal hernia that is not detectable by physical examination, this is referred to as occult [4]. To date, no standardized therapeutic guidelines have been established for occult inguinal hernias accompanied by groin pain [4, 5]. In clinical practice, this diagnosis often results in surgical treatment; however, there is a lack of evidence to support this decision [4–6].

Few studies have reported the prevalence of occult inguinal hernias without any related symptoms. One study observed a prevalence of 16% for occult inguinal hernias when groin ultrasonography was conducted in 100 healthy males [7]. Although contralateral exploration during unilateral laparoendoscopic inguinal hernia repair remains controversial, the HerniaSurge Group International Guidelines for Groin Hernia Management report that the prevelance of occult inguinal hernias ranges from 5 to 58% during this procedure [8–18]. However, the prevelance of occult inguinal hernias and associated groin pain remains unknown.

It is important to recognize that an occult inguinal hernia may not be the true origin of inguinal pain and could merely represent an incidental finding alongside an alternative diagnosis. First, the accuracy of ultrasound is reduced if no palpable hernia is present [19, 20]. Second, given the positive predictive value (PPV) of 70% for ultrasound in detecting occult inguinal hernias, there is a high chance of negative groin exploration, indicating that radiologic findings may not always be accurate [19, 21]. Therefore, it is likely that in some cases, complaints persist or even increase after surgical management [6]. Conversely, delaying treatment could lead to prolonged suffering [22].

At present, knowledge regarding the optimal treatment strategy for patients with groin pain and diagnosis of an occult inguinal hernia is insufficient and may result in unnecessary surgical interventions and inadequate patient counseling. To answer this question, a multicenter randomized controlled trial (RCT), the EFFECT trial, was conducted to compare pain levels and quality of life (QoL) between a watchful waiting (WW) approach and totally extraperitoneal (TEP) repair in this specific patient population. The trial hypothesizes that WW is non-inferior to TEP repair for patients with inguinal pain and an occult inguinal hernia.

## Materials and methods

### Design

The EFFECT trial was a multicenter, non-blinded, RCT that evaluated the non-inferiority of WW compared to TEP repair in patients with groin pain and an occult inguinal hernia. The trial involved eight Dutch hospitals and all hernia surgeons surpassed the learning curve for TEP repair (> 250 procedures). The trial protocol has been previously published [23]. Ethical approval was granted by the regional Medical Ethics Committee (MEC-U, Nieuwegein, the Netherlands) and the local ethics boards of the participating hospitals. The trial was registered in the Dutch Trial Register (ICTRP search portal ID: NL6658).

### Participants

All adult patients presenting at the outpatient clinic with unilateral groin pain (measured on the numeric rating scale [NRS] ≥ 1) without a clinically evident inguinal hernia were screened for eligibility. Patients were deemed eligible if an occult inguinal hernia was present, defined as the absence of clinical features of an inguinal hernia (no visible or palpable groin swelling and a negative Valsalva maneuver). Positive findings of an inguinal hernia on ultrasonography were mandatory for inclusion. No cut-off for the diameter of the hernial defect was applied. To reflect daily practice, ultrasonography was requested in advance by a general practitioner or a surgeon at the outpatient clinic. Patients were excluded if they had a history of groin swelling on the symptomatic side, previous inguinal hernia surgery in the same region, a Body Mass Index (BMI) ≥ 40 kg/m², an American Society of Anesthesiologists (ASA) classification > III, or were unable to complete follow-up with questionnaires (e.g., due to language difficulties).

Written informed consent was obtained from all patients after inclusion. Subsequently, 1:1 randomization was performed by the coordinating investigator of the initiating hospital using the online data management platform. The allocation of treatment was transparent to all the involved parties. Data collection and storage were conducted using a uniform electronic case report form.

### Additional imaging

For baseline comparability, pelvic radiography and MRI of the groin with the Valsalva maneuver were performed. These investigations influenced subsequent management in cases that required different treatment strategies (fractures, malignancy, osteoarthritis, and other relevant conditions), leading to referral and exclusion from the study.

### Intervention

Patients in the WW group were treated with rest, analgesics (NSAIDs), physiotherapy, or a combination of these interventions. This treatment arm was deliberately not standardized because of the expected heterogeneity of the clinical presentation in this group. The treating physician decided which strategy was indicated for each patient, determined the intensity and frequency of rest and/or analgesics, and initiated physiotherapy if considered beneficial. Patients were replaced in case of dropout within 3 months of follow-up.

Patients in the surgical arm underwent TEP repair. The operative technique and perioperative care protocols were standardized across all participating hospitals [23]. All patients underwent surgery under general anesthesia and received a 10 × 15 cm polypropylene mesh without fixation. Follow-up after treatment was performed according to in-hospital protocols. If withdrawal occurred before surgery, the patient left the study and was replaced.

### Outcomes

The primary outcome was the mean difference in NRS between baseline and 3 months of follow-up after treatment, measured at rest and during exercise using the validated EuraHS Quality of Life (EuraHS-QoL) instrument [24].

Secondary outcomes included pain, QoL, patient satisfaction, crossover rate, and PPV of ultrasound. QoL was evaluated using the EuroQol-5D-5L (EQ-5D-5L) questionnaire and questions 4 to 7 of the EuraHS-QoL instrument [25]. The questionnaires were sent by email and completed at baseline, and at 1.5, 3, 6, and 12 months post-intervention. Patient satisfaction was measured at 3 and 12 months of follow-up using a self-designed 11-point Likert scale ranging from 0 to 10 (0 = no satisfaction, 10 = complete satisfaction), as presented in Supplement 2. The crossover rate was represented by the percentage of patients initially receiving WW according to randomization, but crossed over to TEP repair due to persistent pain or a clinically evident inguinal hernia. Crossover to the TEP group from the WW group was only allowed after 3 months of follow-up. The PPV of ultrasonography was calculated using the intraoperative findings as the gold standard.

Other study parameters included baseline characteristics, intraoperative findings, and outpatient clinic observations. Baseline characteristics consisted of sex, ASA, BMI, smoking habit, symptomatic groin side, NRS at the first outpatient clinic consult, and the duration of complaints (in months). The intraoperative presence of an inguinal hernia, classification according to the European Hernia Society (EHS) hernia classification, presence of a lipoma, operative time, conversion to an open procedure, and both intraoperative and postoperative complications were recorded. Outpatient clinic observations encompassed median follow-up time (in days), presence of patient-reported complaints (yes/no), and detection of a palpable inguinal hernia.

### Follow-up

In addition to standard postoperative care, study-related follow-ups evaluating complaints and performing physical examinations were conducted at 3 and 12 months after treatment at the outpatient clinic. Due to the COVID-19 pandemic, hospitals were unable to invite patients to visit outpatient clinics between March 2020 and June 2020. Therefore, telephone consults using the Post-INguinal-repair-Questionnaire (PINQ-PHONE) were used for follow-up [26, 27]. The detection of recurrence using this PINQ-PHONE has been shown to be reliable, with a sensitivity of 100% and a specificity of 86% [26]. Because of the pandemic, both follow-up methods were offered to the patient after this  period, allowing them to choose their preferred method. Follow-up was completed 12 months after the start of the intervention.

### Sample size

The sample size was determined based on the assumption that WW was non-inferior to TEP repair, meaning the mean difference in NRS scores between baseline and the 3-month follow-up in the WW group would not be worse than in the TEP group. Consensus on the minimal clinically important difference in NRS is lacking, but the literature describes a one-point difference as clinically meaningful [28]. In the calculation, a non-inferiority margin of 0.75 points on the NRS was used and it was considered as a continuous variable. The expected variance was estimated using data from 919 patients collected prospectively, where a standard deviation (SD) of 2.3 for the difference in NRS and a correlation of 0.8 between pre-treatment pain and change in pain scores were observed [29]. After correction for baseline pain intensity using analysis of covariance (ANCOVA), the sample size was calculated to be 72 patients per arm [30]. Considering a 10% loss to follow-up, the total sample size was set at 160 patients (80 per arm).

### Statistical analysis

In the published protocol, the proposed test for analyzing the primary outcome was ANCOVA [23]. However, the assumptions of outliers and homoscedasticity of variance were not met, which is why deviations from the protocol were made. To determine non-inferiority, a mixed-effects model was deemed more appropriate as it ensures robustness by accounting for both time and missing values. The non-inferiority margin of the original protocol was maintained (0.75 NRS difference). A 97.5% confidence interval (CI) was used to account for multiple testing.

The secondary continuous outcomes of EuraHS-QoL and EQ-5D-5L were also analyzed with this mixed-effects model correcting for baseline scores (CI 97.5%; *p* < 0.025). EQ-5D-5L index (EQ-index) scores were calculated according to the corresponding Dutch EQ-5D-5L Versteegh value set Version 2.1 (updated 07-04-2021). Non-normally distributed data were compared between the two groups at several follow-up moments using a Wilcoxon rank-sum test. For the comparison of binary categorical variables, the chi-squared test was used.

The primary analysis followed a modified intention-to-treat (mITT) approach. The term mITT was used, because pre-treatment dropouts were excluded from follow-up and they could not be replaced due to premature trial termination. Secondary outcomes were analyzed using both the mITT and the As-Treated (AT) approaches, as crossover from WW to TEP repair was allowed after 3 months of follow-up.

## Results

The study ended prematurely because the target inclusion rate was not achieved during the COVID-19 pandemic. From December 29, 2017, to March 4, 2022, 117 patients were assessed for eligibility. After screening, 18 patients declined randomization for various reasons and were excluded. The study population comprised 99 patients, of whom 51 were allocated to WW and 48 to TEP repair.

### Baseline assessment

**Fig. 1:**
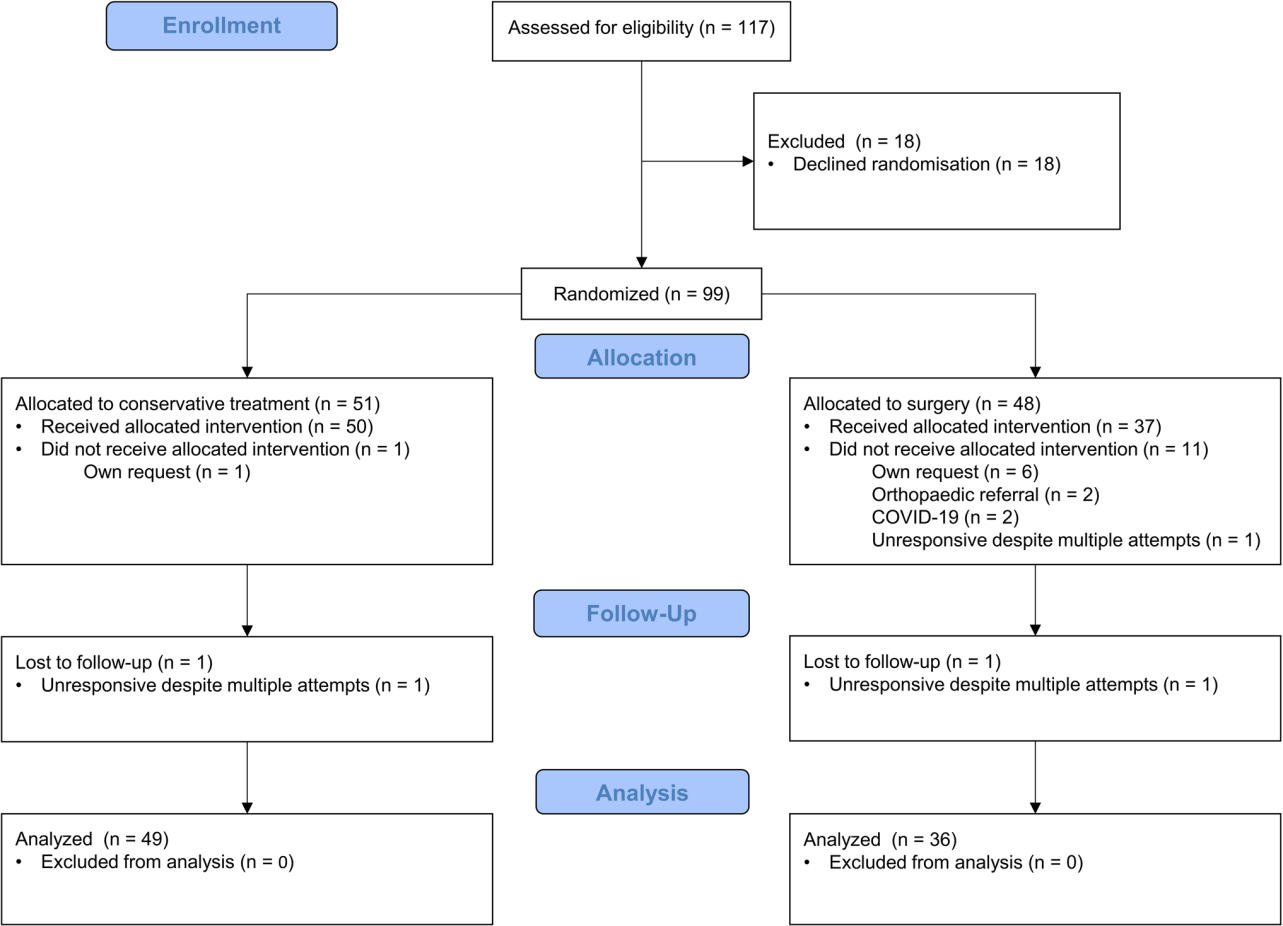
Flowchart of inclusions

In the TEP group, the pelvic X-ray led to referral to an orthopedic surgeon and subsequent treatment in two patients (Fig. 1). The MRI results did not require referral or a different treatment strategy. The difference in size between the two groups can be explained by premature termination of the trial, which prevented the replacement of dropouts. One patient in each treatment arm was lost to follow-up before assessment of the primary endpoint at 3 months because of unresponsiveness despite multiple contact attempts. In total, fourteen patients were excluded after randomization, leaving 85 patients for analysis in the mITT population: 49 in the WW group and 36 in the TEP group. The baseline characteristics are summarized in Table 1.

**Table 1:** Baseline characteristics (*n* = 85)

	WW	TEP
No. of patients	49	36
Sex, *n *(%)		
Male	41 (84)	32 (89)
Female	8 (16)	4 (11)
Age, median (IQR), years	58 (44–67)	54 (41–62)
BMI, mean (SD), kg/m^2^	26 (4)	27 (3)
Current smoker, *n* (%)	8 (16)	4 (11)
Side of complaints, *n* (%)		
Left	20 (41)	18 (50)
Right	29 (59)	18 (50)
ASA, *n* (%)		
I	25 (51)	22 (61)
II	21 (43)	12 (33)
III	3 (6)	2 (6)
Duration of complaints, median (IQR), months	5 (2–12)	4 (2–7)
Baseline pain, *n* (%), NRS		
Mild pain (1–3)	18 (37)	10 (28)
Moderate pain (4–7)	30 (61)	26 (72)
Severe pain (8–10)	1 (2)	0 (0)

*WW* watchful waiting, *TEP* totally extraperitoneal, *IQR* interquartile range, *BMI* body mass index, *SD* standard deviation, *ASA* American Society of Anesthesiologists, *NRS* numeric rating scale

### Intraoperative findings

All 36 patients assigned to the TEP group in the mITT population received the assigned treatment. The intraoperative findings are shown in Table 2. The mean operative time was 21 minutes (SD, 8). No conversions or intraoperative or postoperative complications were observed.

**Table 2:** Intraoperative findings, *n* (%)

Characteristics	TEP (*n* = 36)
No abnormalities	6 (17)
No hernia or lipoma with	
Dilated inguinal ring	2 (6)
Weakness posterior abdominal wall	3 (8)
Dilated inguinal ring and weakness posterior abdominal wall	1 (3)
Lipoma	11 (30)
Inguinal hernia	6 (17)
Inguinal hernia and lipoma	7 (19)
Type of hernia	
Lateral	10 (28)
Medial	2 (6)
Femoral	1 (3)

*TEP* totally extraperitoneal

### Outpatient clinic observations

In the WW group, all 49 patients adhered to the assigned treatment. A palpable hernia was identified in five patients (10%) in the WW group 3 months after treatment *(p* = 0.042). Four of these patients crossed over to TEP repair at that time; one patient had an asymptomatic inguinal hernia and did not undergo surgery. The patient who did not undergo surgery remained asymptomatic after 12 months of follow-up. Table 3 outlines the follow-up visits of the two groups. The results of the AT analyses at the 12-month follow-up are presented in eTable 1 of Supplement 3.

**Table 3:** Outpatient clinic observations at 3 and 12 months in the mITT analysis

Parameters	WW (*n* = 49)	TEP (*n* = 36)	*p* value
**3 months of follow-up**			
No. (%) available for follow-up	44 (90)	34 (94)	
Follow-up time, median (IQR), days	100 (91–129)	100 (91–117)	0.824
Any complaints, *n* (%)	32 (65)	25 (69)	0.937
Palpable inguinal hernia, *n* (%)	5 (10)	0 (0)	0.042*
**12 months of follow-up**			
No. (%) available for follow-up	42 (86)	34 (94)	
Follow-up time, median (IQR), days	397 (378–438)	390 (377–428)	0.562
Any complaints, *n* (%)	18 (37)	15 (42)	0.912
Palpable inguinal hernia, *n* (%)	1 (2)	0 (0)	0.365

* *p* < 0.05

*mITT* modified intention-to-treat, *WW* watchful waiting, *TEP* totally extraperitoneal, *IQR* interquartile range

### Primary outcome

**Fig. 2:**
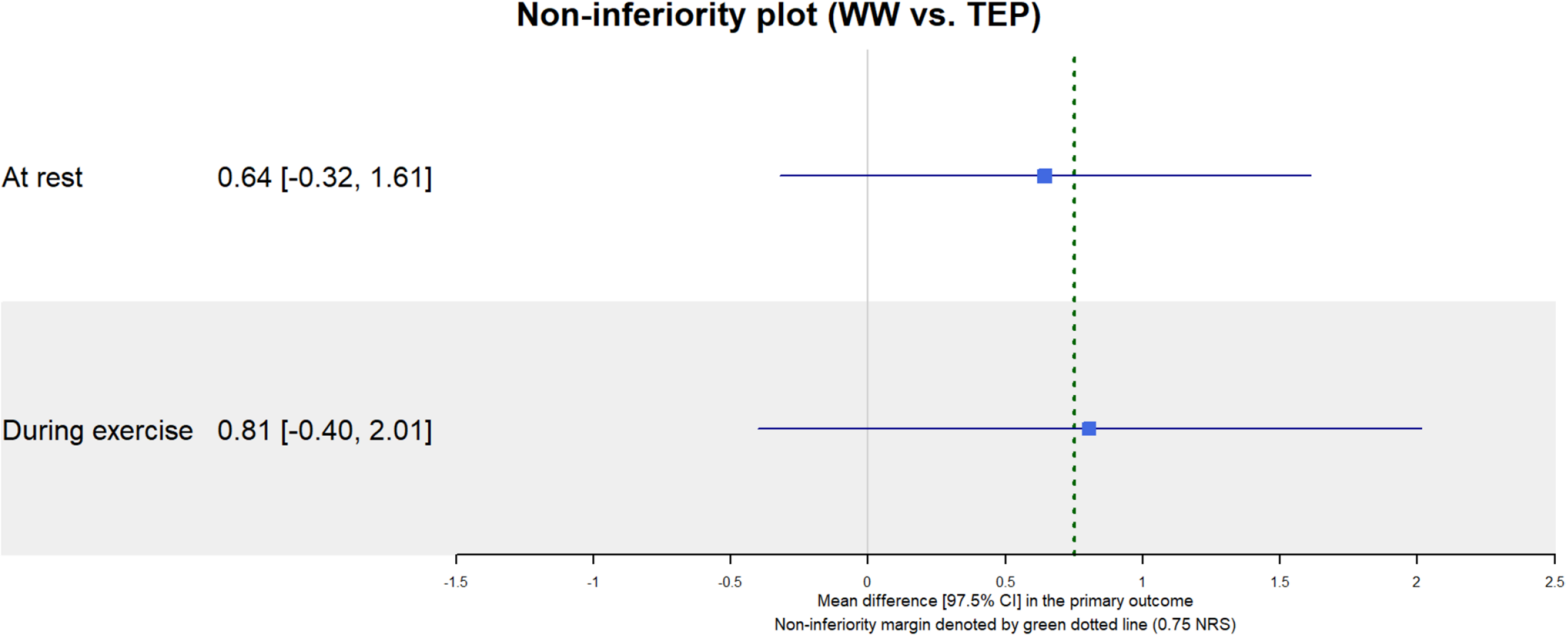
Non-inferiority forest plot. *WW* watchful waiting, *TEP* totally extraperitoneal, *CI* confidence interval, *NRS* numeric rating scale

**Fig. 3:**
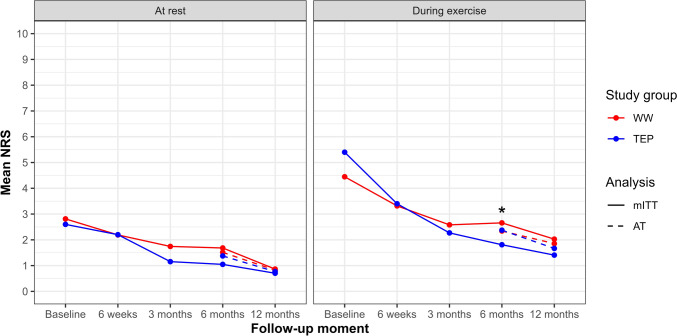
Mean NRS at rest and during exercise. *mITT: *p* = 0.011; *NRS* numeric rating scale, *WW* watchful waiting, *TEP* totally extraperitoneal, *mITT* modified intention-to-treat, *AT*, as-treated

The model reported a mean difference of 0.644 (97.5% CI: − 0.321 to 1.610) for pain at rest and 0.806 (97.5% CI: − 0.402 to 2.014) for pain during exercise. As illustrated in Fig. 2, the upper bounds of the confidence intervals exceed the non-inferiority margin for both pain scores. Figure 3 displays the progression of pain at rest and during exercise over time in both the mITT and AT analyses.

### Other pain-related outcomes and QoL

In the mITT analysis, pain at rest and worst pain felt during the last week were comparable between the groups throughout all follow-up time points. A consistent pattern was observed across the remaining EuraHS-QoL components, all of which assessed pain during some form of activity. These outcomes were similar up to the 3-month follow-up, showed significant differences at 6 months, and became comparable  again at the 12-month follow-up. Similarly, mean EQ-index scores were comparable at all time points except at 6 months. EQ visual analog scale (EQ-VAS) scores remained similar throughout the entire follow-up period. The AT analysis of secondary outcomes showed no significant differences between groups over time. Details on the mean differences and CIs are provided in Supplement 4.

### Patient satisfaction

In the mITT analysis, the median treatment satisfaction score at 3 months was 8.0 (IQR, 7.0–9.0) in the WW group and 8.0 (IQR, 6.8–8.3) in the TEP group (*p* = 0.493). At 12 months, the median score increased to 8.5 (IQR, 8.0–10.0) in the WW group, while it remained unchanged in the TEP group at 8.0 (IQR, 7.0–9.0; *p* = 0.622). Results from the AT analysis at 12 months were similar, with scores of 8.5 (IQR, 8.0–10.0) for WW and 8.0 (IQR, 7.0–9.0) for TEP (*p* = 0.492).

### Crossover

In total, five patients assigned to the WW group crossed over to surgery after the 3-month follow-up time point, resulting in a crossover rate of approximately 10%. Crossover occurred due to persisting complaints (*n* = 1) or a palpable inguinal hernia during physical examination (*n* = 4). For one crossover patient, the transabdominal preperitoneal (TAPP) technique was used instead of TEP, which caused exclusion for the AT analysis. All five patients who crossed over were found to have abnormalities of the abdominal wall. Two had intraoperative lipomas, while the remaining three were diagnosed with inguinal hernias: one lateral, one medial, and one combined obturator and lateral hernia.

### Pain scores of crossovers

The pain scores of the individual patients who crossed over are plotted in eFig. 1 in Supplement 5. The median time from inclusion to crossover was 145 days (IQR, 109–159). In 4 of 5 patients (80%), pain decreased by at least 1 point on the NRS after surgery with a median long-term follow-up of 213 days (IQR, 199–280) postoperatively. One patient experienced persistent pain at rest and increased pain during exercise after surgery. This patient reported that the pain diminished after total hip arthroplasty, which occurred after the 12-month follow-up period.

### PPV ultrasound

To calculate the positive predictive value (PPV) of ultrasound compared to intraoperative observations, any abnormal findings of the abdominal wall observed during surgery—including weakness, a dilated ring, an inguinal hernia, a lipoma, or combined abnormalities—were classified as a true inguinal hernia. This resulted in a PPV of 85% for ultrasound examination.

## Discussion

The EFFECT trial failed to demonstrate non-inferiority of WW compared to TEP repair for pain relief at 3 months post-intervention in patients with groin pain and an occult inguinal hernia. However, this result does not confirm that WW is inferior to TEP repair. Secondary outcomes were comparable between the groups until 3 months. At 6 months  after treatment, pain-related outcomes and QoL favored TEP repair in the mITT population, but by the 12-month study endpoint, these differences in outcomes were no longer observed. The AT analysis showed similar secondary outcomes at both 6 and 12 months. Furthermore, patients were equally satisfied with their assigned treatment. Crossover from WW to surgical repair occurred in 5 out of 49 patients (10%); all of these patients exhibited intraoperative abnormalities of the abdominal wall (lipoma, inguinal hernia, or both). Four of the five (80%) crossover patients experienced pain relief during long-term follow-up. Yet, 15% of patients assigned to the operative arm showed no abnormalities intraoperatively. Therefore, upfront TEP repair carried a risk of overtreatment.

Existing literature provides limited information on patients with inguinal pain and occult inguinal hernias. Only a few cohort studies with small sample sizes have evaluated this population, all of which have shown positive outcomes for conservative management at longer follow-up periods [31–33]. At a median follow-up of 9 months, Corvatta et al. reported that pain was resolved in 54% of their patients (*n* = 98), which occurred spontaneously in 75% of the population, while the remaining 25% required medical treatment [31]. However, this medical treatment was not specified. Melloy et al. observed a higher number of pain-free patients (68%, *n* = 42) after 3 years of non-operative management. Similar to Corvatta et al., they did not clarify what kind of non-operative management was executed, but surprisingly, no participants received physiotherapy [32]. Both studies focused on a WW strategy without comparing pain scores to those of a surgically treated group, limiting the ability to draw definitive clinical conclusions. In contrast, Aly et al. examined the outcomes using the EuraHS-QoL instrument in 32 surgically treated and 31 conservatively treated patients with groin pain and an occult inguinal hernia. The validated EuroHS-QoL instrument used in their study was similar to the method used in the present study [33]. They found no statistically significant differences in outcomes between the two groups [33]. However, detailed information on the follow-up period and specific treatment strategies is lacking. These limitations inhibit adequate comparison with our results.

The ultrasound-detected occult inguinal hernia could be either the true cause of complaints, an incidental finding alongside a different underlying cause, or a false-positive finding reflecting normal anatomy. If patients with inguinal pain and an occult inguinal hernia experience pain due to a genuine or early-stage inguinal hernia, it is likely that they will require surgery over time. Notably, the intraoperative findings of this study demonstrated abnormalities of the abdominal wall in 85% of the operated groins, which comprised weakness of the posterior abdominal wall, dilation of the inguinal ring, lipoma, inguinal hernia, or a combination of the aforementioned abnormalities. These findings are consistent with those of van Hout et al., who studied a population similar to ours and reported that 87% of their patients (*n* = 179) had a hernia defect, lipoma, or weak posterior wall [6]. These findings may help explain why surgery is effective in case of an occult inguinal hernia and associated inguinal pain.

The role of additional imaging in the workup of occult inguinal hernias is questionable given that our study observed a PPV of 85% for detecting occult inguinal hernias by ultrasound. Preoperative ultrasound findings may have demonstrated true abdominal wall defects or weaknesses, sliding lipomas, or false-positive findings representing normal anatomic variation of the inguinal canal. As a result, unnecessary surgeries were still performed, with six patients (15%) undergoing negative explorations. A possible explanation for the reduced accuracy of ultrasound is that it is susceptible to subjectivity and heavily relies on operator skills. For example, during the Valsalva maneuver, some fatty tissue may slide into the inguinal ring or minor bulging of the abdominal wall could potentially be interpreted as an inguinal hernia [7]. Insights from a recent study suggest that addressing ultrasound variability could benefit from a standardized protocol, as the optimal approach for diagnosing occult inguinal hernias is unknown [3]. A recent Delphi-based consensus [3] highlighted key recommendations for diagnosing occult inguinal hernias, such as the use of dynamic ultrasound and that reports should include hernia contents and defect size. Providing the radiologist with clinical details, especially  symptoms and surgical history, can also improve diagnostic accuracy [3]. When ultrasound was analyzed in conjunction with clinical judgment, Light et al. reported a PPV of 73%, which was comparable to the 85% observed in our study [20].

The WW strategy for occult inguinal hernias is essentially the same as the approach used for delayed surgery. This is exemplified by the 12-year follow-up of the INCA trial, which concluded that over the long term, three-quarters of WW-assigned patients crossed over to surgery, and this occurred more frequently and significantly earlier in patients with mildly symptomatic hernias than in those with asymptomatic hernias [34]. WW has proven effective in patients aged ≥ 50 years, because the risk of incarceration is low and surgery for asymptomatic or mildly symptomatic inguinal hernias may lead to chronic postoperative inguinal pain (CPIP) [35, 36]. Identifying which patients may benefit from surgical repair while under WW may help guide clinical decision-making in this population. If the pain resolved over time and no clinically detectable inguinal hernia was observed, it was most likely caused by another condition. Pain unresponsive to conservative management may serve as a reason for crossover to TEP repair, provided that alternative diagnoses are unlikely.

This is the first RCT to compare WW and TEP repair in patients with groin pain and an occult inguinal hernia, which represents a major strength of the study. The premature ending of the study due to the COVID-19 pandemic, which consequently caused failure to achieve the required sample size, is a limitation. Therefore, our results should be interpreted with caution. The exclusion of 12-month pain scores as part of the primary outcome is a limitation, as this could have provided a more comprehensive understanding of the long-term effects of both treatments. Furthermore, crossover from the TEP group to WW prior to surgery was not possible in this trial. Tracking the outcomes of patients who dropped out from the TEP group might have yielded additional insights. Future studies should aim to assess long-term results (beyond 12 months) in this study population, with particular emphasis on crossover rates and recurrence of complaints.

This study did not demonstrate non-inferiority of WW compared to TEP repair in this distinct patient population, which can partly be explained by the limited sample size. However, this does not confirm that WW results in worse outcomes compared to TEP repair, since secondary outcomes remained comparable through the 3-month follow-up. Moreover, TEP repair was associated with a potential risk of overtreatment, as 15% of patients exhibited no intraoperative abnormalities. Therefore, a WW strategy for at least 3 months may help identify which patients could benefit from TEP repair. During this period, a clinically detectable inguinal hernia may emerge, indicating the need for surgery, or the pain, if due to another cause, may resolve. For patients who prefer not to wait or do not respond to conservative management, TEP repair should be considered. The EFFECT trial highlights the importance of individualized decision-making based on patient preferences, clinical context, and long-term management goals.

## Supplementary Information

Below is the link to the electronic supplementary material.

Supplementary file1 (DOCX 165 KB)
