# Supplementary material for: Watchful waiting versus totally extraperitoneal (TEP) hernia repair for occult inguinal hernia and pain (EFFECT trial)—a multicenter, non-inferiority, randomized controlled trial
Source: Surg Endosc. 2025 May 22;39(7):4266–76. doi: 10.1007/s00464-025-11681-w (PMC12222410; doi:10.1007/s00464-025-11681-w)

**Online Supplementary Material**

*Table of contents*

1. Supplement 1: Author Collaboration
2. Supplement 2: Patient satisfaction on 11-point Likert scale
3. Supplement 3: eTable 1 Outpatient clinic observations at 3 and 12 months in the AT analysis
4. Supplement 4: eTable 2 Secondary outcomes by follow-up timepoint in mITT and AT analyses
5. Supplement 5: eFig. 1 Pain scores (NRS) at rest and during exercise plotted individually for all crossover patients

**Supplement 1**

*EFFECT study group*

Richtje R Meuzelaar, Egbert-Jan MM Verleisdonk, Anandi HW Schiphorst, Floris PJ den Hartog, Pieter J Tanis, Ine PJ Burgmans, Marleen M Roos, Wouter J Bakker, Coen V van Hessen, Floris BM Sanders (Diakonessenhuis, Utrecht, The Netherlands); Paul M Verheijen (Meander Medisch Centrum, Amersfoort, The Netherlands); Frank WH Kloppenberg (Treant Zorggroep, Hoogeveen, The Netherlands); Huib A Cense (Rode Kruis Ziekenhuis, Beverwijk, The Netherlands); Mirjam Stuijvenberg (St. Antonius Ziekenhuis, Nieuwegein, The Netherlands); Caroline ME Contant (Maasstad Ziekenhuis, Rotterdam, The Netherlands); Henk F van Stel, Rogier KJ Simmermacher, Arno W Hoes, Rebecca K Stellato, Geert W Frederix (UMC Utrecht, The Netherlands), Andre de Vries (Amsterdam UMC, locatie AMC, The Netherlands)

**Supplement 2**

Patient satisfaction on 11-point Likert scale in Dutch and English

**Bent u tevreden over de behandeling die u is toegewezen in deze studie?** Vul een cijfer in, waarbij geldt: 0= ‘erg ontevreden’, 10= ‘zeer tevreden’

Tevreden 0 1 2 3 4 5 6 7 8 9 10

**Are you satisfied with the treatment assigned to you in this study? *Please enter a number, where: 0 = 'very dissatisfied', 10 = 'very satisfied'***

Satisfaction 0 1 2 3 4 5 6 7 8 9 10

**Supplement 3**

**eTable 1** Outpatient clinic observations at 3 and 12 months in the AT analysis

| **Parameters** | **WW (n = 44)** | **TEP (n = 40)** | ***p*-value** |
| --- | --- | --- | --- |
| **3 months of follow-up** |  |  |  |
| No. (%) available for follow-up | 39 (89) | 38 (95) |  |
| Follow-up time, median (IQR), days | 99 (91 – 127) | 101 (91 – 120) | 0.948 |
| Any complaints, n (%) | 27 (61) | 29 (73) | 0.444 |
| Palpable inguinal hernia, n (%) | 1 (2) | 3 (8) | 0.165 |
| **12 months of follow-up** |  |  |  |
| No. (%) available for follow-up | 38 (86) | 37 (93) |  |
| Follow-up time, median (IQR), days | 399 (380 – 442) | 388 (374 – 428) | 0.264 |
| Any complaints, n (%) | 16 (36) | 17 (43) | 0.817 |
| Palpable inguinal hernia, n (%) | 1 (2) | 0 (0) | 0.314 |

AT, as-treated; WW, watchful waiting; TEP, totally extraperitoneal; IQR, interquartile range

**Supplement 4**

**eTable 2a** Mean NRS differences and 97.5% CIs for secondary EuraHS-QoL outcomes across the different follow-up time points in the mITT analysis

| **Follow-up** | **Mean NRS difference** | **97.5% CI** | ***p*-value** |
| --- | --- | --- | --- |
| **Pain at rest** | | | |
| 6 weeks | -.024 | -1.062 – 1.014 | .958 |
| 3 months | .644 | -.321 – 1.610 | .133 |
| 6 months | .610 | -.435 – 1.656 | .189 |
| 12 months | .227 | -.766 – 1.221 | .606 |
| **Pain during exercise** | | | |
| 6 weeks | .512 | -.766 – 1.790 | .366 |
| 3 months | .806 | -.402 – 2.014 | .133 |
| 6 months | 1.484 | .183 – 2.786 | **.011*** |
| 12 months | 1.181 | -.082 – 2.443 | .036 |
| **Worst pain felt during the last week** | | | |
| 6 weeks | 1.025 | -.136 – 2.185 | .048 |
| 3 months | .626 | -.461 – 1.713 | .194 |
| 6 months | 1.067 | -.107 – 2.240 | .041 |
| 12 months | .737 | -.389 – 1.863 | .141 |
| **Restrictions during indoor activities** | | | |
| 6 weeks | .483 | -.545 – 1.512 | .290 |
| 3 months | .792 | -.171 – 1.755 | .065 |
| 6 months | 1.262 | .234 – 2.290 | **.006*** |
| 12 months | .896 | -.104 – 1.895 | .044 |
| **Restrictions during outdoor activities** | | | |
| 6 weeks | .426 | -.893 – 1.746 | .466 |
| 3 months | .798 | -.454 – 2.049 | .151 |
| 6 months | 2.032 | .690 – 3.374 | **<.001*** |
| 12 months | 1.271 | -.029 – 2.572 | .028 |
| **Restrictions during sports** | | | |
| 6 weeks | .149 | -1.658 – 1.956 | .852 |
| 3 months | .636 | -1.073 – 2.344 | .399 |
| 6 months | 1.967 | .170 – 3.765 | **.014*** |
| 12 months | .730 | -1.068 – 2.528 | .358 |
| **Restrictions during heavy labour** | | | |
| 6 weeks | 1.023 | -.590 – 2.635 | .153 |
| 3 months | .657 | -.876 – 2.191 | .333 |
| 6 months | 2.004 | .398 – 3.610 | **.005*** |
| 12 months | 1.539 | -.051 – 3.129 | .030 |

* *p* < 0.025

NRS, numeric rating scale; CI, confidence interval; EuraHS-QoL, EuraHS Quality of Life; mITT, modified intention-to-treat

**eTable 2b** Mean EQ-index differences and CIs across the different follow-up time points in the mITT analysis

| **Follow-up** | **Mean EQ-index difference** | **97.5% CI** | ***p*-value** |
| --- | --- | --- | --- |
| 6 weeks | -0.014 | -.081 – .052 | .630 |
| 3 months | -0.027 | -.091 – .037 | .341 |
| 6 months | -0.104 | -.172 – -.035 | **<.001*** |
| 12 months | -0.019 | -.085 – .047 | .518 |

* *p* < 0.025

EQ-index, EQ-5D-5L questionnaire index score; CI, confidence interval; mITT, modified intention-to-treat

**eTable 2c** Mean EQ-VAS differences and CIs across the different follow-up time points in the mITT analysis

| **Follow-up** | **Mean EQ-VAS difference** | **97.5% CI** | ***p*-value** |
| --- | --- | --- | --- |
| 6 weeks | -1.131 | -9.062 – 6.800 | .748 |
| 3 months | -1.580 | -9.017 – 5.857 | .632 |
| 6 months | -2.977 | -11.188 – 5.233 | .414 |
| 12 months | 2.265 | -5.381 – 9.911 | .504 |

EQ-VAS, EQ visual analog scale; CI, confidence interval; mITT, modified intention-to-treat

**eTable 3a** Mean NRS differences and 97.5% CIs for secondary EuraHS-QoL outcomes across the different follow-up time points in the AT analysis

| **Follow-up** | **Mean NRS difference** | **97.5% CI** | ***p*-value** |
| --- | --- | --- | --- |
| **Pain at rest** | | | |
| 6 weeks | -.256 | -1.261 – .750 | .567 |
| 3 months | .150 | -.800 – 1.100 | .722 |
| 6 months | .146 | -.882 – 1.174 | .749 |
| 12 months | .124 | -.866 – 1.113 | .778 |
| **Pain during exercise** | | | |
| 6 weeks | .270 | -1.001 – 1.541 | .631 |
| 3 months | -.052 | -1.273 – 1.168 | .923 |
| 6 months | .680 | -.627 – 1.987 | .241 |
| 12 months | .742 | -.545 – 2.029 | .194 |
| **Worst pain felt during the last week** | | | |
| 6 weeks | .714 | -.424 – 1.852 | .158 |
| 3 months | .285 | -.797 – 1.368 | .552 |
| 6 months | .085 | -1.079 – 1.249 | .869 |
| 12 months | .662 | -.471 – 1.795 | .188 |
| **Restrictions during indoor activities** | | | |
| 6 weeks | .194 | -.823 – 1.211 | .666 |
| 3 months | .160 | -.809 – 1.128 | .709 |
| 6 months | .503 | -.529 – 1.534 | .272 |
| 12 months | .731 | -.284 – 1.745 | .105 |
| **Restrictions during outdoor activities** | | | |
| 6 weeks | .061 | -1.262 – 1.385 | .917 |
| 3 months | -.155 | -1.427 – 1.118 | .783 |
| 6 months | .927 | -.430 – 2.284 | .124 |
| 12 months | .868 | -.465 – 2.201 | .142 |
| **Restrictions during sports** | | | |
| 6 weeks | -.125 | -1.914 – 1.664 | .874 |
| 3 months | -.394 | -2.113 – 1.324 | .603 |
| 6 months | .537 | -1.278 – 2.351 | .503 |
| 12 months | .262 | -1.563 – 2.087 | .745 |
| **Restrictions during heavy labour** | | | |
| 6 weeks | .363 | -1.251 – 1.978 | .611 |
| 3 months | .108 | -1.443 – 1.658 | .875 |
| 6 months | .587 | -1.035 – 2.208 | .413 |
| 12 months | .972 | -.645 – 2.588 | .175 |

NRS, numeric rating scale; CI, confidence interval; EuraHS-QoL, EuraHS Quality of Life; AT, as-treated

**eTable 3b** Mean EQ-index differences and CIs across the different follow-up time points in the AT analysis

| **Follow-up** | **Mean EQ-index difference** | **97.5% CI** | ***p*-value** |
| --- | --- | --- | --- |
| 6 weeks | .000 | -0.066 – 0.066 | .998 |
| 3 months | -.012 | -0.075 – 0.052 | .678 |
| 6 months | -.056 | -0.124 – 0.012 | .064 |
| 12 months | -.007 | -0.073 – 0.060 | .819 |

EQ-index, EQ-5D-5L questionnaire index score; CI, confidence interval; AT, as-treated

**eTable 3c** Mean EQ-VAS differences and CIs across the different follow-up time points in the AT analysis

| **Follow-up** | **Mean EQ-VAS difference** | **97.5% CI** | ***p*-value** |
| --- | --- | --- | --- |
| 6 weeks | -.144 | -7.807 – 7.520 | .966 |
| 3 months | -.920 | -8.192 – 6.351 | .775 |
| 6 months | -.062 | -8.051 – 7.926 | .986 |
| 12 months | 2.461 | -5.107 – 10.029 | .463 |

EQ-VAS, EQ visual analog scale; CI, confidence interval; AT, as-treated

**Supplement 5**

**eFig. 1** Pain scores (NRS) at rest and during exercise plotted individually for all patients whom crossed over. NRS, numeric rating scale


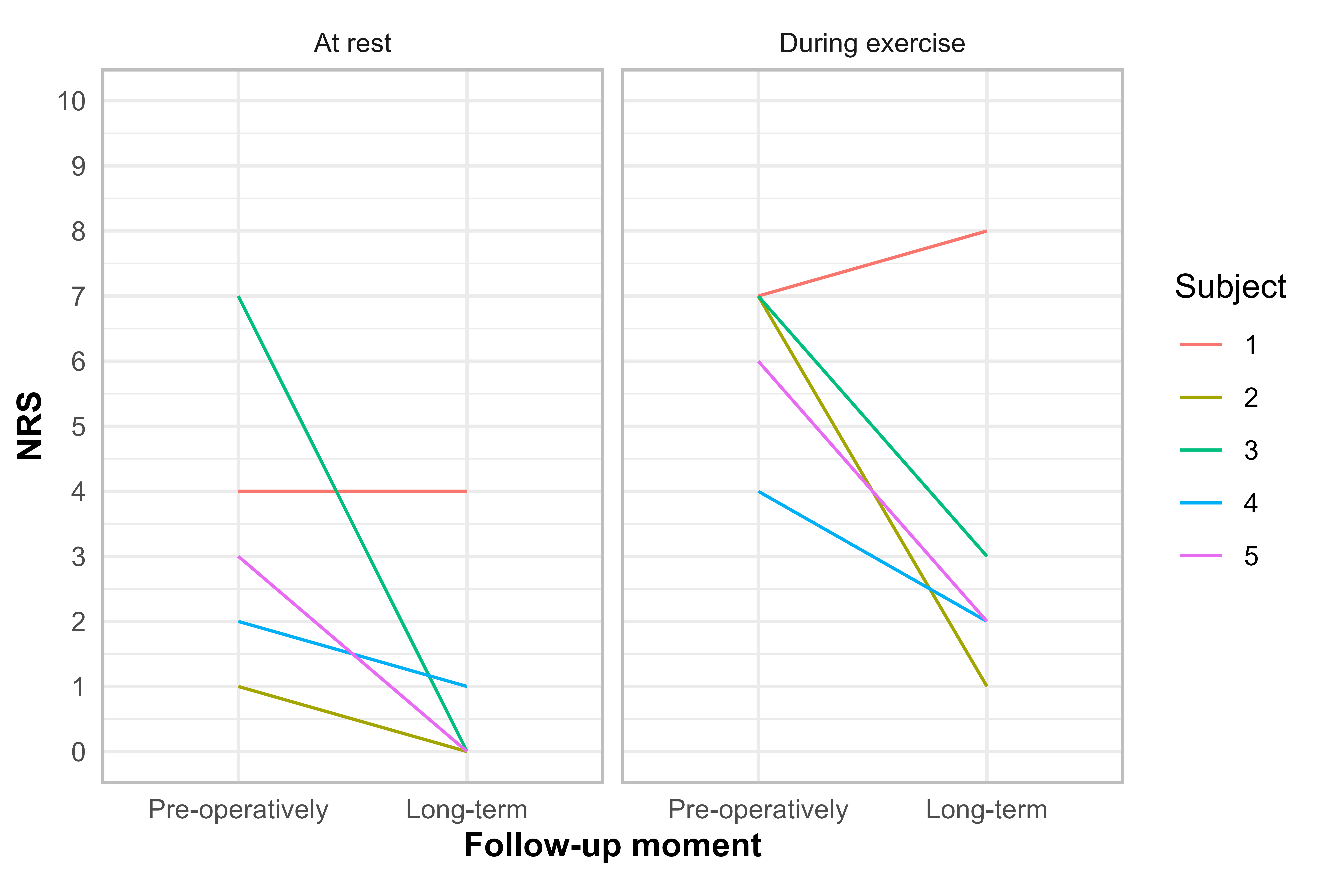

Supplement: Supplementary file 1 — Supplementary file1 (DOCX 165 KB) [file 464_2025_11681_MOESM1_ESM.docx]
